# Charcot-Marie-Tooth Type 2B: A New Phenotype Associated with a Novel *RAB7A* Mutation and Inhibited EGFR Degradation

**DOI:** 10.3390/cells9041028

**Published:** 2020-04-21

**Authors:** Paola Saveri, Maria De Luca, Veronica Nisi, Chiara Pisciotta, Roberta Romano, Giuseppe Piscosquito, Mary M. Reilly, James M. Polke, Tiziana Cavallaro, Gian Maria Fabrizi, Paola Fossa, Elena Cichero, Raffaella Lombardi, Giuseppe Lauria, Stefania Magri, Franco Taroni, Davide Pareyson, Cecilia Bucci

**Affiliations:** 1 Department of Clinical Neurosciences, Fondazione IRCCS Istituto Neurologico Carlo Besta, 20133 Milan, Italy; paola.saveri@istituto-besta.it (P.S.); chiara.pisciotta@istituto-besta.it (C.P.); raffaella.lombardi@istituto-besta.it (R.L.); giuseppe.lauriapinter@istituto-besta.it (G.L.); 2 Department of Biological and Environmental Sciences and Technologies, University of Salento, 73100 Lecce, Italy; maria.deluca@unisalento.it (M.D.L.); veronica.nisi@libero.it (V.N.); roberta.romano@unisalento.it (R.R.); 3 Functional Neuromotor Rehabilitation Unit, IRCCS ICS Maugeri Spa-SB, Scientific Institute of Telese Terme, 82037 Telese Terme (Benevento), Italy; giuseppe.piscosquito@icsmaugeri.it; 4 MRC Centre for Neuromuscular Diseases, UCL Queen Square Institute of Neurology, London WC1N 3BG, UK; m.reilly@ucl.ac.uk; 5 Department of Neurogenetics, The National Hospital for Neurology and Neurosurgery, UCL Institute of Neurology, London WC1N 3BG, UK; james.polke@nhs.net; 6 Department of Neurosciences, Biomedicine and Movement Sciences, University of Verona, 37134 Verona, Italy; tiziana.cavallaro@aovr.veneto.it (T.C.); gianmaria.fabrizi@univr.it (G.M.F.); 7 Department of Pharmacy, School of Medical and Pharmaceutical Sciences, University of Genova, 16132 Genova, Italy; fossa@difar.unige.it (P.F.); cichero@difar.unige.it (E.C.); 8 Department of Biomedical and Clinical Sciences Luigi Sacco, University of Milan, 20157 Milan, Italy; 9 Unit of Medical Genetics and Neurogenetics, Department of Diagnostics and Technology, Fondazione IRCCS Istituto Neurologico Carlo Besta, 20133 Milan, Italy; stefania.magri@istituto-besta.it (S.M.); franco.taroni@istituto-besta.it (F.T.)

**Keywords:** RAB7A, Charcot–Marie–Tooth disease type 2B, CMT2B, peripheral sensory neuropathy, NGF, RAB7, mutations, axons, lysosomes, autophagy, neurite outgrowth, endocytosis, EGFR

## Abstract

The rare autosomal dominant Charcot-Marie-Tooth type 2B (CMT2B) is associated with mutations in the *RAB7A* gene, involved in the late endocytic pathway. CMT2B is characterized by predominant sensory loss, ulceromutilating features, with lesser-to-absent motor deficits. We characterized clinically and genetically a family harboring a novel pathogenic *RAB7A* variant and performed structural and functional analysis of the mutant protein. A 39-year-old woman presented with early-onset walking difficulties, progressive distal muscle wasting and weakness in lower limbs and only mild sensory signs. Electrophysiology demonstrated an axonal sensorimotor neuropathy. Nerve biopsy showed a chronic axonal neuropathy with moderate loss of all caliber myelinated fibers. Next-generation sequencing (NGS) technology revealed in the proband and in her similarly affected father the novel c.377A>G (p.K126R) heterozygous variant predicted to be deleterious. The mutation affects the biochemical properties of RAB7 GTPase, causes altered interaction with peripherin, and inhibition of neurite outgrowth, as for previously reported CMT2B mutants. However, it also shows differences, particularly in the epidermal growth factor receptor degradation process. Altogether, our findings indicate that this *RAB7A* variant is pathogenic and widens the phenotypic spectrum of CMT2B to include predominantly motor CMT2. Alteration of the receptor degradation process might explain the different clinical presentations in this family.

## 1. Introduction

Autosomal dominant axonal Charcot–Marie–Tooth disease type 2B (CMT2B) largely overlaps with hereditary sensory-autonomic neuropathies (HSANs) as it is characterized by predominant sensory loss, high frequency of ulcer formations and amputations, with variable motor deficits [1,2,3,4,5,6,7,8,9,10]. Affected patients show severe distal sensory loss particularly for pain and touch, reduced-to-absent deep tendon reflexes, foot deformities, and sometimes distal wasting and weakness mainly in lower limbs [1,2,3,4,5,7,8,9,10]. The disease typically starts during the second or third decade [10] and runs a slowly progressive course [11,12]. Males have a higher occurrence of ulcers [4], suggesting a difference in disease severity according to gender as for HSAN I [13].

CMT2B is caused by heterozygous mutations in *RAB7A*, with five mutations (L129F, K157N, N161T/I, V162M) reported in patients from eleven families [1,2,3,4,5,7,8,9,10], all displaying the characteristic ulcero-mutilating phenotype with variable motor involvement (Table 1).

Pathomechanisms, whereby *RAB7A* mutations lead to CMT2B, are a matter for debate and investigation. RAB7A, hereafter referred to as RAB7, is a member of the Rab family of small GTPases involved in the regulation of vesicular trafficking between early endosomes and lysosomes, controlling transport to the degradative compartments in the endocytic pathway and lysosome biogenesis [14]. RAB7 modulates the Endoplasmic Reticulum (ER) morphology by controlling the ER homeostasis and ER stress [15]. Crosstalk occurring at mitochondria-lysosome contact sites regulated by Rab7 has also been recently demonstrated [16].

Although ubiquitously expressed, RAB7 has specific functions in neurons, where it regulates retrograde axonal trafficking and signaling of neurotrophin receptors, as well as neurite outgrowth [17,18]. Furthermore, RAB7 regulates cell migration by influencing integrin trafficking and vimentin assembly [19] and cortical neurons’ migration during development [20].

Interestingly, RAB7 has specific effectors in neurons as co-immunoprecipitates with the neurotrophin receptor TrkA (Tropomyosin-receptor-kinase A) and interacts directly with the intermediate filament protein peripherin [18,21]. Therefore, it is not surprising that mutations in *RAB7* cause a disease restricted to neurons, although it is unclear why sensory neurons are so selectively vulnerable.

Previous biochemical characterization of four CMT2B-causative RAB7 mutants showed increased dissociation rate constant (K_off,_) for nucleotides and lower GTPase activity per binding event [22,23,24]. Overexpression of these mutant proteins inhibits neurite outgrowth in several cell lines [25,26]. Furthermore, these RAB7 mutant proteins display stronger interaction with some RAB7 effector proteins, including RILP (RAB-interacting lysosomal protein), required for lysosomal transport towards the microtubule organizing center (MTOC) by inducing dynein-dynactin motors recruitment [27].

RAB7 and RILP control degradation of the epidermal-growth-factor receptor (EGFR), a member of the receptor tyrosine-kinase family involved in regulating cell proliferation, survival, differentiation and migration [28,29]. Importantly, EGF seems also to have important neurotrophic functions [30,31].

Previous experiments on EGFR degradation obtained on cells transiently or stably transfected with CMT2B-causing RAB7 mutants gave conflicting results: transient expression of these mutants demonstrated normal or increased EGFR degradation [22,23] while stable transfection revealed inhibition [32].

Here, we report a family with a novel CMT2B phenotype with motor predominance and absence of ulcers and mutilations, carrying a novel pathogenic *RAB7* variant (c.377A>G, p.K126R) which is absent in global databases, affects a highly conserved amino-acid in the GTPase domain of Rab7, is predicted to be pathogenic by *in silico* analysis, and is transmitted as an autosomal dominant trait. We performed extensive biochemical and functional studies, which confirmed its pathogenic role.

## 2. Materials and Methods

### 2.1. Patients

**Figure 1:**
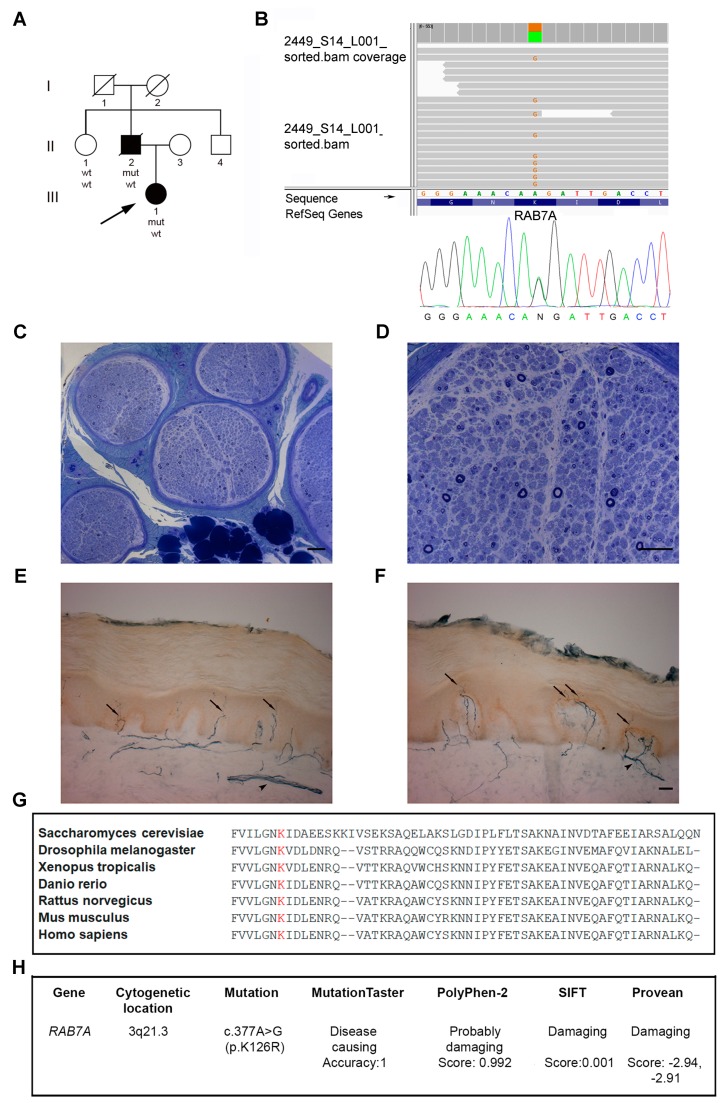
Pedigree, DNA sequencing, nerve, and skin biopsy of the proband. (**A**) Family pedigree. (**B**) Next-Generation Sequencing and Sanger chromatogram of the proband with the heterozygous c.377A>G (p.K126R) variant in the *RAB7* gene. (**C**,**D**) Sural nerve biopsy from the 18-year-old proband. (**C**) Semithin section stained with toluidine blue showing a uniform and moderate loss of fibers. (**D**) At higher magnification, no degenerating or regenerating fibers were observed. Scale bars: C = 100 μm; D = 50 μm. Skin biopsies from the 38-year-old proband (**E**) and a 52-year-old healthy female individual (**F**), taken at the medial side of the proximal phalanx of the index finger. (**E**,**F**) Immunostaining with anti-protein gene product 9.5 antibodies (PGP9.5) showed a minimal reduction of the intraepidermal nerve fiber (IENF) density in the proband (**E**) as compared to control (**F**). Arrows indicate intra-epidermal nerve fibers and arrowheads indicate dermal nerve bundles. Scale bars: E, F = 50 μm. (**G**) The CLUSTAL multiple sequence alignment by MUSCLE (3.8) shows the conservation of lysine amino-acid at position 126 during evolution [35]. (**H**) *In silico* analysis: RAB7^K126R^ variant was predicted to be pathogenic by major online programs.

We evaluated clinically and electrophysiologically (standard procedures) one healthy and two affected family members (Figure 1A). Informed consent was obtained for all procedures from study participants.

The index case underwent a biopsy of sural nerve biopsy which was processed for histological and ultrastructural examination [33]. 3-mm skin biopsies were taken (from shoulder and the lateral aspect of the proximal phalanx of the index finger) for fibroblast culture, immunohistochemistry, and intraepidermal nerve fiber (IENF) count [34].

### 2.2. Gene Sequence Analysis

The proband’s DNA was analyzed by Next Generation Sequencing (NGS) technology with a probe-based customized panel for CMT and related disorders (Illumina Nextera Rapid Capture Custom kit, Illumina Inc., San Diego, CA, USA), (Tables S1–S3). Sequencing was performed using the NGS MiSeq sequencer (Illumina Inc.). The entire *RAB7* gene-targeted region (6 coding exons and 25 bp of flanking introns) was sequenced by NGS with a depth of coverage >20×. The sequence variant was confirmed in proband and her father by the Sanger method (Figure 1B).

### 2.3. Mutagenesis and Plasmid Construction

Most constructs used in this study have been described previously [14,22,29]. RAB7^K126R^ mutant was constructed using the QuickChange XL Site-Directed Mutagenesis Kit (Stratagene, San Diego, CA, USA). The oligonucleotides used to generate the mutant were 5′-GTGTTGGGAAACAGGATTGACCTCG-3′ and 5′-CGAGGTCAATCCTGTTTCCCAACAC-3′. The mutant RAB7^K126R^ plasmids were obtained using RAB7^wt^ cDNA previously cloned in pcDNA3-2xHA or pET-16b vector in frame with DNA coding for hemagglutinin (HA) or poly-His tag, respectively.

### 2.4. Antibodies

Western blotting analysis (WB): anti-HA (1:500, sc-805), anti-peripherin (1:500, sc-28539) and anti-vinculin (1:10000, sc-25336) were from Santa Cruz Biotechnology, Santa Cruz, CA, USA; anti-actin (1:5000, ab-8224) and anti-tubulin (1:6000, clone B512), were from Sigma-Aldrich, St-Louis, MO, USA, while anti-EGFR (1:2000, 20-ES04) was from Fitzgerald, Concord, MA, USA and anti-RILP (1:400, 13574–1-AP) from Proteintech, Rosemont, IL, USA. Immunofluorescence analysis: anti-early endosome antigen 1 (EEA1, 1:1000, ab70521, Abcam), anti-HA (1:500, ab9110, Abcam), anti-EGFR (1:100, 20-ES04, Fitzgerald). Secondary antibodies conjugated to fluorochromes for immunofluorescence or horseradish peroxidase (HRP) were from Invitrogen (Carlsbad, CA, USA), Santa Cruz Biotechnology or Fitzgerald. Immunohistochemistry: myelin basic protein (MBP, 1:100, ab7349, Abcam), anti-peripherin (1:1000, ab4666, Abcam), anti-vasoactive intestinal peptide (VIP, 1:400, ab22736, Abcam), anti-neurofilament 200 (NF-H, 1:400, N0142, Sigma-Aldrich), anti-PGP9.5 (1:500, MCA4750GA, Bio-Rad, Hercules, CA, USA), anti-EGFR (1:100, 20-ES04, Fitzgerald). Secondary antibodies Alexa Fluor-conjugated (Jackson ImmunoResearch, Cambridge, UK) were employed.

### 2.5. Cells and Transfection

Neuro2A and NCI H1299 cells, fibroblasts from CMT2B patients and control were grown in DMEM supplemented with 10% or 15% FBS, 2 mM L-glutamine, 100 U/mL penicillin and 10 mg/mL streptomycin.

Transfection was performed using Metafectene PRO reagent (Biontex, München, DE) according to manufacturer’s instructions. Cells were analyzed 24 h after transfection.

### 2.6. Western Blotting and Co-Immunoprecipitation

Cells were processed for SDS-PAGE and WB as previously described [22]. Frozen sural nerves were pulverized and sonicated in lysis buffer (95 mM NaCl, 25 mMTris-HCl, pH 7.4, 10 mM EDTA, 2% SDS, and protease inhibitors) and lysates were subjected to WB [20].

Densitometry analysis was performed using the NIH ImageJ program or Gene Tools from Syngene and normalized to appropriate loading controls signal intensity. For immunoprecipitation, we used the anti-HA affinity gel (Ezview Red Anti-HA E6779, Sigma-Aldrich) according to the manufacturer’s instruction.

### 2.7. Confocal Immunofluorescence Microscopy

Cells grown on 12-mm round glass coverslips were fixed, permeabilized with 0.1% Triton X-100 for 5 min at room temperature and incubated with primary and secondary antibodies as previously described [21]. Cells were viewed with a Zeiss LSM700 confocal microscope. Zen 2011 software (Carl Zeiss, Oberkochen, Germany) was used for image capture and to calculate the weighted colocalization coefficient of EGFR and EEA1.

Skin biopsies were sectioned (50 μm) using a standard cryostat (Bio-Optica, Milan, Italy). Immunohistochemistry assays were performed following a standard free-floating protocol. Fluorescent images were acquired with Leica TSC SP8 confocal microscope (Leica Microsystems, Wetzlar, Germany).

### 2.8. Nucleotide Dissociation and GTPase Assay

Nucleotide exchange assay and determination of K_off_ were performed as previously reported [36]. The GTPase assay was performed as previously described [37]. GTP hydrolysis was quantified as a GDP signal relative to GDP+GTP signal [37].

### 2.9. EGFR Degradation Assay

To measure EGFR degradation cells were treated and processed as previously described [22].

### 2.10. Neurite Outgrowth Assay

Differentiation of Neuro2A was triggered by serum withdrawal as previously described [25]. Cells were co-transfected with the pEGFPC1 vector (to express GFP and visualize the entire cell) and with a plasmid for expression of HA-tagged RAB7^wt^ or mutant proteins, fixed, permeabilized and stained [19]. For each experiment, to determine the percentage of cells bearing neurites (>50 μm), approximately 100 transfected cells were counted in at least 10 randomly chosen visual fields. We compared the number of cells bearing neurites longer than 50 μm between control cells and cells expressing HA-tagged RAB7^wt^ and mutant proteins.

### 2.11. Real-Time PCR

Total RNA was isolated using the RNeasy Micro kit (Qiagen, Hilden, Germany) and retrotranscription was made using SuperScript II Reverse Transcriptase (Invitrogen) according to the manufacturer’s instructions.

Quantitative real-time PCR was carried out with Power SYBR Green (Applied Biosystems, Foster City, USA) using Applied Biosystems 7900HT Fast Real-time PCR System. The primers used were GAPDH forward 5′-GGTGGTCTCCTCTGACTTCAACA-3′ and reverse 5′-GTTGCTGTAGCCAAATTCGTTGT-3′; EGFR forward 5′-GGCAGGAGTCATGGGAGAA-3′ and reverse: 5′-GCGATGGACGGGATCTTAG-3′ from Eurofins Genomics (Ebersberg, Germany). The thermal profile used: 1 cycle of 2 min at 50 °C; 1 cycle of 10 min at 95 °C; 40 cycles of 15 s at 95 °C, 1 min at 55 °C; 1 cycle of 15 s at 95 °C and 15 s at 60 °C. The relative expression level was calculated using the comparative C_T_ method and expressed as “fold change.” The relative quantification was considered significant when there was a minimum of two-fold change.

### 2.12. Molecular Modeling Studies

Wild type RAB7 structure was retrieved from the Protein Data Bank (pdb code 1T91) and used to generate the model of RAB7^K126R^ protein (Pymol, The PyMOL Molecular Graphics System, Version 1.2r3pre, Schrödinger, LLC). Molecular dynamics simulations were performed within MOE (MOE, Chemical Computing Group Inc., Montreal H3A 2R7 Canada).

### 2.13. Statistical Analysis

Data were statistically analyzed using Student’s t-test or χ^2^ test (* *p* < 0.05, ** *p* < 0.01 and *** *p* < 0.001). Experiments were performed at least in triplicate.

## 3. Results

### 3.1. Patients

Patients’ clinical features are reported in Table 2.

The proband (III-1) (Figure 1A), a 39-year-old female, at age 14 years started complaining of calf muscle cramps, progressive walking difficulties, distal muscle wasting and weakness, recurrent ankle sprains and balance issues. At age 31 years, she underwent bilateral foot surgery for Achilles tendon lengthening and toes straightening. Afterward, she started wearing ankle-foot orthoses (AFOs). She reported no symptoms in upper limbs and minimal sensory loss in the feet. Neurological examination showed steppage gait, mild wasting, and weakness of intrinsic hand muscles (MRC 4+), moderate distal atrophy in lower limbs, severe paresis of toe and foot dorsiflexors (MRC 0), and moderate foot plantar-flexion weakness (MRC 3). She had mild pinprick sensory loss in the feet, slightly reduced vibration sense at the great toe, and absent ankle jerks. She had neither ulcers nor amputations. CMTES was 9/28.

Nerve conduction studies (NCS) showed no motor responses in the peroneal nerves, decreased compound muscle action potential (CMAP) amplitude in the tibial nerve (0.4 mV), the reduced amplitude of right ulnar nerve (5.9 µV) sensory action potential (SAP), unobtainable sural nerve SAP, with normal findings at other examined motor and sensory nerves in upper limbs. Nerve conduction velocities (NCVs) were preserved. EMG showed severe neurogenic changes in lower limb distal muscles.

Nerve biopsy (age 18) showed a chronic axonal neuropathy, with moderate and diffuse loss of myelinated fibers of all calibers (Figure 1C); residual fibers had normal myelin sheaths. There were no signs of ongoing degeneration or regeneration (Figure 1D). Electron microscopy confirmed the histologic findings disclosing some collagen pockets or denervated, flattened, Schwann cell processes indicating loss of unmyelinated fibers.

Skin IENF density (age 38) was 5.9 fibers/mm, showing only a minimal difference (Figure 1E) with a gender-matched control (6.4 fibers/mm, Figure 1F).

Her father (II-2) (Figure 1A) had symptom onset at age 11 years, manifesting progressive foot-drop and walking difficulties, with later occurrence of wasting and weakness of hand muscles, difficulties in doing buttons, and balance loss. He did neither undergo foot surgery nor complain of positive sensory symptoms. Neurological examination at age 55 years showed *pes cavus*, ataxic and steppage gait, positive Romberg sign, moderate hand tremor, severe distal limb wasting and weakness, with intrinsic hand muscles MRC = 1–2 and complete loss of foot movements, absent ankle jerks, reduced position sense at the great toes, and vibration sense at the feet. Neither ulcers nor amputations were present. CMTES was 11/28 and CMTNS 17/36. NCS was consistent with a severe axonal motor-sensory polyneuropathy: the absence of sensory and motor responses in lower limbs, markedly reduced amplitude of the median CMAP (0.1 mV), ulnar (0.8 µV) and radial (4.4 µV) SAPs. He died of acute leukemia a few months later.

Proband’s aunt (II-1) (Figure 1A) had no history of neuropathy and normal examination and NCS. The grandparents were reported to have no symptoms and died in their 60s (I-2) or 70s (I-1); the uncle (II-4) was asymptomatic and refused clinical examination and genetic testing.

### 3.2. Identification of a Novel RAB7 Mutation Causing CMT2B

NGS analysis revealed a heterozygous missense variant in *RAB7*, c.377A>G (p.K126R), in proband and affected father which was absent in the unaffected aunt and the major gene variant databases (Single Nucleotide Polymorphism database 150, 1000 Genomes, Exome Variant Server, Exome Aggregation Consortium, Genome Aggregation Database) (Table S2). The variant, confirmed in both patients by Sanger sequencing (Figure 1B), is located in a highly conserved amino-acid in the GTPase domain (Figure 1G) and predicted to be deleterious by *in silico* analysis (Figure 1H).

### 3.3. Characterization of the Biochemical Properties of the RAB7^K126R^ Mutant Protein

**Figure 2:**
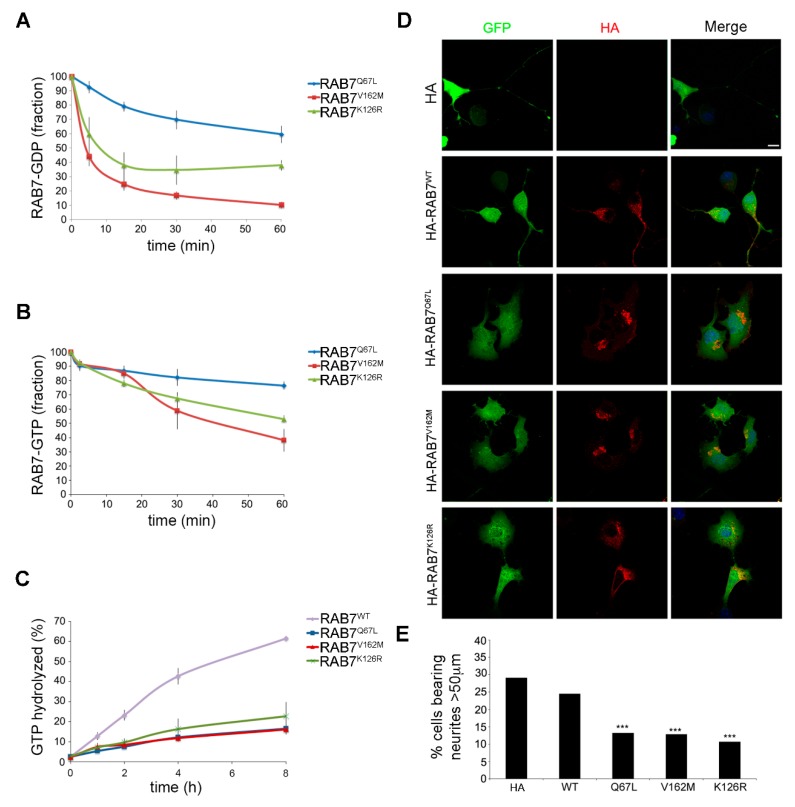
Biochemical and biological properties of the RAB7^K126R^ mutant protein. (**A**,**B**) Dissociation of guanine nucleotides from RAB7^K126R^, RAB7^Q67L^, and RAB7^V162M^. Purified proteins were incubated with [^3^H]GDP (**A**) or [^3^H]GTP (**B**), then a 100× excess of cold competitor (GDP or GTP) was added and dissociation of nucleotide was monitored for 1h to calculate K_off_. (**C**) The measure of GTPase activity of RAB7^wt^, RAB7^K126R^, RAB7^Q67L^, and RAB7^V162M^ purified proteins per binding event. Proteins were loaded with [^32^P]GTP and then GTP hydrolysis was monitored during 8 h adding 100× fold excess of unlabeled GTP. (**D**,**E**) To analyze the effect of RAB7^K126R^ on neurite outgrowth, Neuro2A cells were co-transfected with the indicated HA-tagged RAB7^wt^ and mutant expressing constructs and with the pEGFPC1 plasmid to visualize entire cell and incubated for 24 h in serum-free medium to induce cell differentiation. (**D**) Confocal microscopy images of a field of cells per condition are shown. Scale bar = 10 μm. (**E**) Quantification of neurite outgrowth in Neuro2A cells expressing different RAB7^wt^ and mutant proteins. Neurites were defined as processes longer than 50 μm. The percentage of cells bearing neurites was calculated as the number of cells with neurites divided by the total number of cells. We compared the number of cells bearing neurites longer than 50 μm between control cells (cells transfected with pEGFPC1 and HA empty plasmid) and cells expressing RAB7^wt^ and mutant proteins. We show a statistical analysis of three independent experiments (χ^2^ test, *** *p* < 0.001).

To study the biochemical properties of the RAB7^K126R^ mutant, we analyzed the dissociation rate constants (K_off_) for GDP and GTP. We bacterially expressed His-tagged RAB7^Q67L^ (a constitutively active mutant, impaired in GTP hydrolysis that displays nucleotide K_off_ similar to RAB7^wt^), RAB7^V162M^ (a previously characterized CMT2B mutant) and the new RAB7^K126R^ mutant and purified them by affinity chromatography. Then, we determined the K_off_ for GDP and GTP measuring radioactivity loss over time (Figure 2A,B). As expected, RAB7^Q67L^ showed GDP and GTP K_off_ similar to previously published data on RAB7^wt^ [22,36] while the RAB7^V162M^ CMT2B mutant protein displayed higher K_off_ for both GTP and GDP, as previously reported [22]. Interestingly, also the RAB7^K126R^, similarly to the other CMT2B-causing mutant proteins, exhibited K_off_ values significantly higher for GDP and GTP (Figure 2A,B).

We also measured GTPase activity per binding event by loading the purified His-tagged RAB7^wt^, RAB7^Q67L^, RAB7^V162M^ and RAB7^K126R^ with ^32^P-GTP (Figure 2C). The RAB7 estimated hydrolysis rate constant was consistent with previous reports [22,36]. Moreover, as previously reported, the RAB7^Q67L^ and the CMT2B-causing RAB7^V162M^ mutants showed lower GTPase activity [22]. Notably, also the RAB7^K126R^ showed impaired GTPase activity per binding event similarly to the RAB7^V162M^ mutant (Figure 2C).

Altogether these data demonstrate that the RAB7^K126R^ mutant displays higher nucleotide K_off_ (particularly high for GDP) and impaired GTP hydrolysis per binding event, similarly to previously analyzed CMT2B-causing mutant proteins [22,23,24].

### 3.4. Expression of the RAB7^K126R^ Mutant Inhibits Neurite outgrowth in Neuro2A Cells

To analyze the impact of the RAB7^K126R^ mutant on a neuronal-specific process, we studied the effect on neurite outgrowth in neuroblastoma Neuro2A cells, which were transfected with the pEGFP plasmid to visualize the cells and therefore, the neurites. Cells were co-transfected with the HA empty plasmid (control cells) or with plasmids encoding HA-tagged RAB7^wt^, RAB7^Q67L^, RAB7^V162M^, or RAB7^K126R^. After transfection and differentiation, the cells were processed for immunofluorescence analysis using anti-HA antibodies to identify transfected cells and scored for the presence of neurites longer than 50 μm (Figure 2D). About 30% of control cells had long neurite after serum withdrawal and similar values were obtained also in cells overexpressing RAB7^wt^ (Figure 2E). In contrast, as expected, expression of the RAB7^Q67L^ or RAB7^V162M^ mutant resulted in a strong neurite outgrowth inhibition (Figure 2E), consistent with previous results [25]. Similarly, RAB7^K126R^ mutant expression considerably inhibited neurite outgrowth, by about 60% (Figure 2E).

### 3.5. Analysis of Peripherin and RILP, two known Downstream RAB7 Effectors

**Figure 3:**
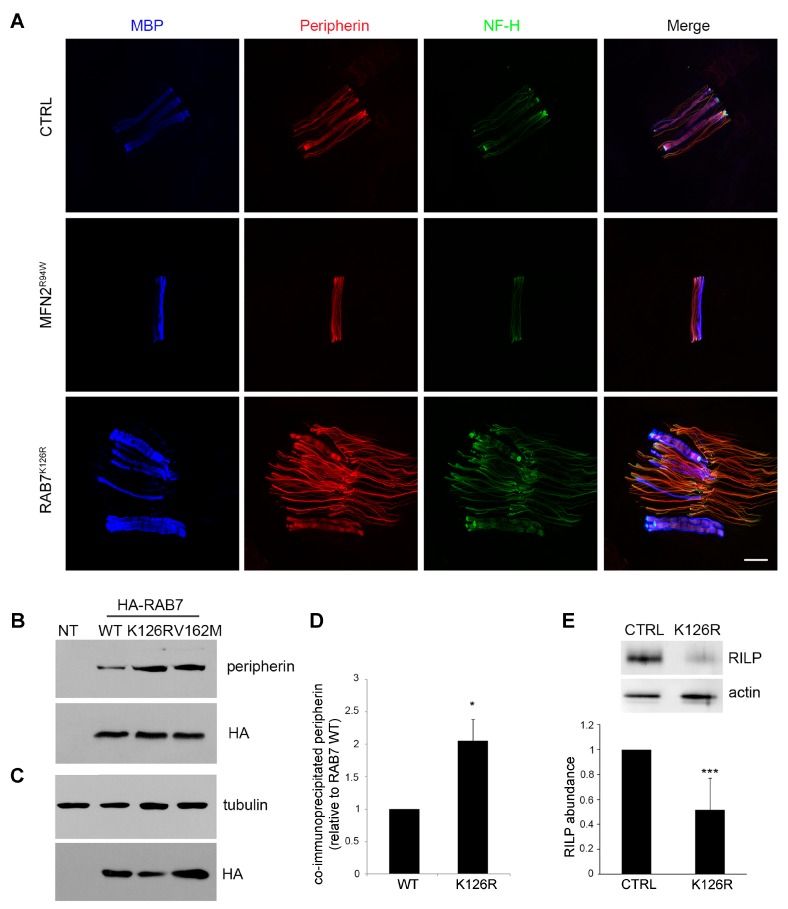
Analysis of two RAB7 interactors: peripherin and RAB-interacting lysosomal protein (RILP). (**A**) Immunohistochemical staining was performed on skin biopsy section using antibodies direct to peripherin, neurofilament-200, and MBP (myelinated nervous fibers) in the proband, in a patient with a mutation in *MFN2* (axonal neuropathy), and in a healthy control. Scale bar = 20 μm in all images. (**B**) HA-tagged RAB7^wt^ and mutant proteins were expressed in Neuro2A cells, as indicated, immunoprecipitated with an anti-HA antibody and subjected to WB using anti-peripherin and anti-HA antibodies. (**C**) Total extracts of Neuro2A cells, overexpressing the indicated proteins, were analyzed using anti-HA and anti-tubulin antibody to evaluate expression levels of the proteins. (**D**) Quantification of immunoprecipitated peripherin. Values are the mean of three independent experiments ± s.e.m.. The intensities were quantified by densitometry, normalized against the amount of RAB7^wt^ or RAB7^K126R^, and plotted as a percentage of the intensities obtained using RAB7^wt^ (set to 1). Data were analyzed statistically using Student’s t-test. Values of cells expressing RAB7^K126R^ were found to be significantly different from the values of cells expressing RAB7^wt^ (* *p* < 0.05). (**E**) Lysates of the sural nerve from a healthy individual (CTRL) and proband carrying the RAB7^K126R^ mutation were analyzed by immunoblotting using anti-RILP and anti-actin antibodies. The intensities of the RILP bands were quantified using Gene Tools from Syngene and normalized against actin. The intensity of the RILP band in CTRL cells has been set to 1. Data represent the mean ± s.e.m. of six independent experiments. Statistical analysis was performed using Student’s t-test. *** *p* < 0.001.

RAB7 interacts with peripherin, an intermediate filament protein expressed mainly in peripheral nerves, and CMT2B-causing mutants display a stronger interaction compared to RAB7^wt^ [21]. Immunohistochemical study for peripherin in association with anti-MBP and anti-NF-H antibodies showed a markedly increased expression of peripherin and NF-H in the proband compared to a CMT2A patient (MFN2^R94W^, axonal neuropathy control) and a healthy control (Figure 3A).

To investigate how the RAB7^K126R^ mutant interacts with the peripherin, we performed a co-immunoprecipitation experiment (Figure 3B,C). Neuro 2A cells were transfected with the empty plasmid or with constructs encoding HA-tagged RAB7^wt^, RAB7^K126R^, and RAB7^V162M^ and then lysed. HA-tagged proteins were immunoprecipitated and subjected to WB using anti-HA and anti-peripherin antibodies. As expected, peripherin was co-immunoprecipitated by HA-tagged RAB7^wt^ (Figure 3B). The RAB7^V162M^ mutant was able to co-immunoprecipitate peripherin more efficiently, in accordance with previous data [21]. Similarly, peripherin was more efficiently co-immunoprecipitated by the RAB7^K126R^ mutant as compared to RAB7^wt^ (Figure 3B–D).

As RAB7, in association with RILP, controls the late endocytic pathway [21], we decided to analyze RILP abundance in proband’s sural nerve lysate. Similarly to what described for the disease-causing RAB7^N161T^ mutant in an immunostaining experiment [2], the amount of RILP was reduced by almost half (Figure 3E).

### 3.6. Expression of the RAB7^K126R^ Mutant Inhibits Ligand-Induced EGFR Degradation

**Figure 4:**
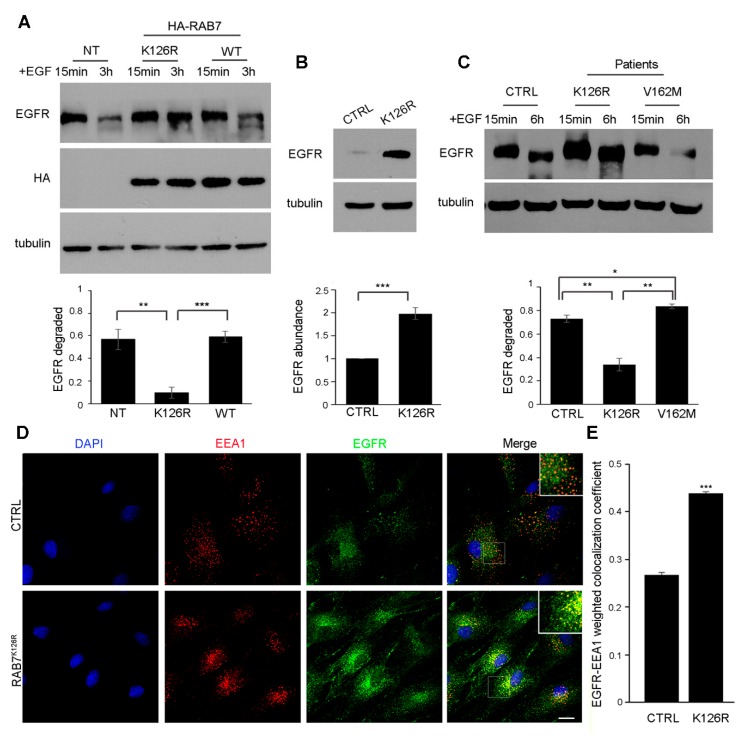
Expression of RAB7^K126R^ inhibits ligand-induced EGFR degradation affecting EGFR intracellular trafficking. (**A**) NCI-H1299 cells overexpressing HA-RAB7^K126R^ or HA-RAB7^wt^ were treated with cycloheximide for 1 h and stimulated for 15 min or 3 h with EGF. Cells were then lysed and analyzed by WB using a specific anti-EGFR antibody to detect undegraded EGFR, and anti-HA and anti-tubulin antibodies to verify RAB7 overexpression and equal loading, respectively. The amount of EGFR degraded at 3 h was quantified and plotted as a percentage of the respective intensity at 15 min set to 1. The error bars represent the s.e.m. of three independent experiments. According to a Student’s t-test, * *p* < 0.05; ** *p* < 0.01. (**B**) Lysates of dermal fibroblasts from a healthy individual (CTRL) and CMT2B patients carrying the RAB7^K126R^ mutation were analyzed by immunoblotting using anti-EGFR and anti-tubulin antibodies. The intensities of the EGFR bands were quantified using NIH ImageJ and normalized against tubulin. The intensity of the EGFR band in CTRL cells has been set to 1. Data represent the mean ± s.e.m. of six experiments. Student’s t-test, *** *p* < 0.001. (**C**) Fibroblasts derived from control and CMT2B patients were incubated with cycloheximide and stimulated with EGF for the indicated times. Cell lysates were analyzed by immunoblotting with antibodies against EGFR and tubulin. Densitometric analysis was performed with NIH ImageJ normalizing against tubulin. Data represent the mean ± s.e.m. of at least three experiments. Student’s t-test, * *p* < 0.05; ** *p* < 0.01. (**D**) Dermal fibroblasts from CTRL and the CMT2B proband carrying the RAB7^K126R^ mutation incubated with cycloheximide and stimulated with EGF for 3 h were subjected to immunofluorescence analysis using specific anti-EGFR (green) and anti-EEA1 (red) antibodies followed by the appropriate secondary antibodies. Scale bar = 20 μm. (**E**) Data represent the mean ± s.e.m. of at least 50 cells of three independent experiments. Statistical analyses were performed using Student’s t-test with control fibroblasts as a referring sample. *** *p* < 0.001.

To establish if the RAB7^K126R^ mutant affects EGFR degradation, a cellular process normally regulated by RAB7 [28] (Figure S1), we performed an EGFR degradation assay (Figure 4A). Quantification revealed that, in agreement with previous data [22,23], about 60% of EGFR was degraded in control cells and in cells expressing RAB7^wt^ (Figure 4A). In contrast, the expression of RAB7^K126R^ strongly inhibited degradation that was significantly different from RAB7^wt^ (Figure 4A). Thus, with respect to EGFR degradation, the RAB7^K126R^ seems to behave differently from the previously discovered CMT2B-causative mutant proteins as a transient expression of these mutants increased EGFR degradation [22,23].

### 3.7. EGFR Amount, Degradation and Intracellular Distribution are Altered in Patient Fibroblasts Carrying the RAB7^K126R^ Mutation

To validate data obtained by transiently expressing the CMT2B-causing RAB7 mutant proteins we performed the assay in skin fibroblasts from the proband and a control. We evaluated first the total EGFR amount in the absence of EGF stimulation. Interestingly, in fibroblasts carrying the RAB7^K126R^ mutation the amount of EGFR was higher compared to control cells (Figure 4B), while EGFR transcript quantification by RT-PCR revealed no significant change in EGFR mRNA levels (data not shown), thus suggesting a difference in EGFR degradation. Therefore, we performed an EGFR degradation assay and, notably, we found that EGFR degradation was strongly inhibited in fibroblasts carrying the RAB7^K126R^ mutation as compared to control cells (Figure 4C). In contrast, EGFR degradation was increased in fibroblasts carrying the V162M mutation (Figure 4C), consistently with previous data [22].

Altogether, these data suggest that the accumulation of EGFR in RAB7^K126R^ is mainly due to impaired protein degradation.

To determine whether the impaired EGFR degradation is due to trafficking alteration we performed immunofluorescence analysis of patient and control fibroblasts using specific antibodies against EGFR and a marker of the early endosomes EEA1 (Figure 4D). Interestingly, EGFR intracellular distribution differed between control and patient cells: in patient cells, the amount of EGFR colocalizing with EEA1 was strongly increased, suggesting impaired trafficking of EGFR to late endocytic organelles (Figure 4E).

### 3.8. EGFR Amount is Altered in Patient Tissues

**Figure 5:**
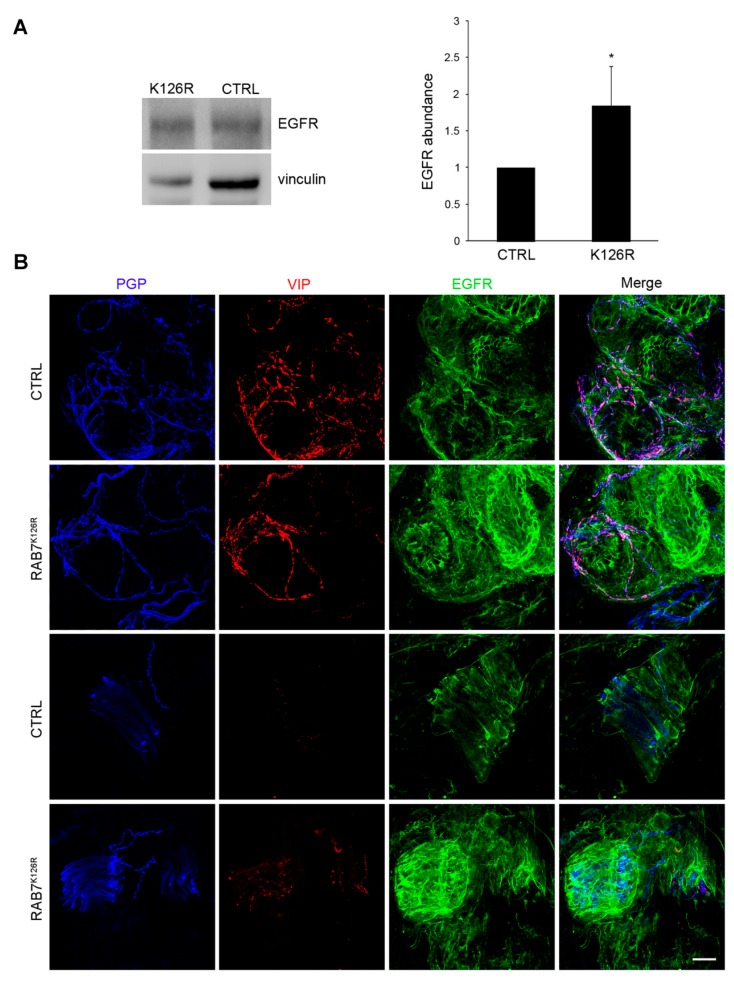
Expression of EGFR in proband sural nerve and skin biopsy sections. (**A**) Sural nerves from the proband and a gender-matched healthy control were lysed and EGFR protein expression was analyzed in both samples by WB using a specific anti-EGFR antibody. Antibody against vinculin was employed as a loading control. Densitometric analysis was performed with Gene Tools from Syngene. Data represent the mean ± s.e.m. of four experiments. The intensity of the EGFR band in CTRL cells has been set to 1. Statistical analysis was performed using Student’s t-test. * *p* < 0.05. (**B**) Confocal microscopy study of a skin biopsy from the proband and a healthy control using a specific anti-EGFR antibody. Nervous fibers are labeled with PGP9.5 (pan-axonal marker) and VIP (sympathetic cholinergic fibers’ marker) antibodies. Scale bar = 20 μm in all images.

We performed WB of sural nerve lysates from the proband and a gender-matched healthy control and we found an almost twofold increase in EGFR expression (Figure 5A), in agreement with *in vitro* results. EGFR expression was also studied in skin biopsy sections: a qualitative analysis of confocal images, stained using antibodies against EGFR, PGP9.5 (pan-axonal marker), and VIP (sympathetic cholinergic fibers’ marker), showed a widespread receptor overexpression in the proband, including areas around nerve fibers (Figure 5B).

### 3.9. RAB7^K126R^ Computational Model

**Figure 6:**
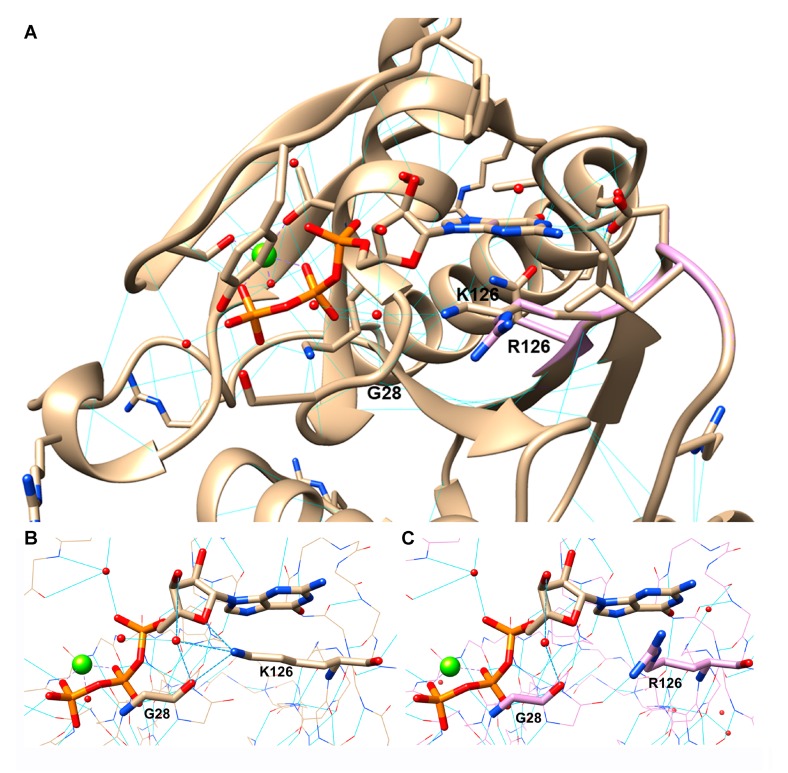
Structural insights into the disease-causing RAB7^K126R^ mutant. (**A**) Comparison between the wild type (pdb code 1T91) and the virtual model of the mutated protein, colored in light brown and light pink, respectively. The RAB7^K126R^ mutation is located in the GTPase domain. (**B**,**C**) The H-bond interactions performed by K126 (**B**) or R126 (**C**), GTP, a conserved water molecule and G28. The protein is colored in light brown (**B**) or light pink (**C**). Bold dashed blue lines highlight the specific H bond net performed by residue 126, GTP, G28, and water. Light blue lines indicate H-bonds. Magnesium ion is represented as a green sphere and water molecules are represented as red spheres.

Residue 126 is located in the GTPase binding site, where the lysine side chain forms two H-bonds with the nucleotide. The first H-bond is made with the ribose ring oxygen, while the second one is an H-bond bridging the amino group with the hydroxyl group on position 4 of the sugar, via a conserved water molecule (Figure 6A). Interestingly, this H-bond bridge involves glycine 28 too, contributing to stabilize not only GTP inside its pocket but also the pocket secondary structure. According to the 3D model, the arginine side chain is unable to perform this H-bonds network, being differently oriented due to the bigger volume of its terminal guanidine group. Thus, RAB7^K126R^ mutation, as previously reported,^33^ seems to reduce the GTP anchoring inside the protein and consequently to determine an impaired RAB7 functionality. Short molecular dynamics simulations performed on both wild type and mutated GTP bound complexes confirm these observations (Figure 6B,C).

## 4. Discussion

CMT2B has long been considered mainly a sensory neuropathy with a large overlap with the HSANs. Only five *RAB7* mutations have been reported, all involving the GTP binding domain and associated with a mainly sensory phenotype.

We identified and characterized a novel missense variant (p.K126R) in *RAB7* associated with predominantly motor CMT2 in the two affected family members. Notably, both subjects had remarkable muscle wasting and weakness with only minor sensory signs, in keeping with CMT2 with motor predominance, never linked previously to *RAB7* mutations. All previous families showed ulcers and amputations, with variable motor involvement (Table 1). Lower occurrence of ulcers is reported in CMT2B females [4], but the motor predominant phenotype was shown also by the father, ruling out a gender effect.

The p.K126R variant affects, like the other CMT2B mutations, the RAB7 GTP-binding domain and its pathogenicity is supported by the involved site, segregation analysis, and *in silico* predictions. The lysine 126 is very conserved not only in RAB7 during evolution (Figure 1G) but also in many other GTPases as this amino-acid is part of the switch II region; it is one of the amino-acids forming the guanine binding pocket and it is a crucial amino-acid residue involved in the hydrogen bond with the nucleotide ribose oxygen [38]. Importantly, mutations in this amino-acid residue are predicted to have an increased rate of dissociation of the nucleotides favoring the active GTP-bound state [38]. Despite arginine shares with lysine basic and charged features, it impairs the GTP pocket architecture. The bulkier side chain of arginine exceeds lysine volume and is thus unable to perform the same specific interactions with GTP and some amino acid residues (water bridging with glycine 28) in the surrounding pocket (Figure 6B,C).

We carried out a series of functional experiments to demonstrate the RAB7^K126R^ pathogenic role and look for differences with other RAB7 mutants, to explain the distinctive predominantly motor phenotype. Our studies confirmed that the mutation affects a series of RAB7 properties, very similar to previously characterized mutants, but with at least one important difference.

First, biochemical data indicate that the mutant protein has an increased K_off_ for both nucleotides and particularly high for GDP (Figure 2A), inhibiting also GTPase activity per binding event (Figure 2C), similarly to previous uncovered CMT2B-causing RAB7 mutant proteins [22,23,24]. Interestingly, the corresponding mutation in the *HRAS* gene, a gene encoding a member of the RAS superfamily of small GTPases as the *RAB7* gene, is associated with the Costello syndrome, a disease causing an increased risk of developing tumors in different organs further supporting pathogenicity of this variant [39].

Second, expression of RAB7^K126R^ strongly inhibits neurite outgrowth, compared to the other CMT2B-causing RAB7 mutants (Figure 2D) [25,26]. As the onset of CMT2B occurs in the second or third decade, similarly to many other CMT types, development appears to be unaffected. However, the mutant proteins can rather impact on axonal regeneration, considering that this process recapitulates all the stages of neuronal differentiation [40]. Indeed, the detrimental action of RAB7 mutants could be counteracted by other factors during development, but with age, these factors could become less effective and a decrease in regeneration capabilities may contribute to the progressive axonal loss underlying clinical manifestations.

Third, the RAB7^K126R^ mutant protein interacts more strongly with the intermediate filament peripherin (Figure 3B–D), as for already reported *RAB7* mutations. Peripherin is important for neuronal morphology, maturation, and differentiation, but also axonal regeneration [41,42]. RAB7 affects peripherin assembly [21] and altered RAB7-peripherin interaction could impair axonal regeneration thus contributing to nerve degeneration. Notably, we found increased labeling of peripherin in skin biopsy where numerous peripherin-positive filamentous structures were seen in contrast to the few seen in controls (Figure 3A).

With respect to both findings, it is noteworthy that the nerve biopsy showed—at least for this single sensory nerve—no evidence of regenerating clusters despite the moderate loss of myelinated fibers (Figure 1D).

Fourth, RAB7^K126R^ mutant protein strongly reduces RILP downstream effector levels (Figure 3E), similarly to RAB7^N161T^ [2]. Notably, in previous functional studies, siRNA-mediated RILP depletion was sufficient to cause strong effects on EGFR degradation and the biogenesis of late endosomes [27,43].

Fifth, our data demonstrate also that expression of the RAB7^K126R^ mutant protein causes inhibition of EGFR degradation in contrast to previously studied CMT2B-RAB7 mutants [22,23]. Indeed, expression of the RAB7^L129F^, RAB7^K157N^, RAB7^N161T^, and RAB7^V162M^ mutants did not reveal any inhibition of EGFR degradation but rather a small and reproducible, though not statistically significant, increase [22,23]. Furthermore, analysis of EGFR degradation in RAB7^V162M^ fibroblasts revealed an increase of EGFR degradation compared to control fibroblasts (Figure 4C), while, expression of RAB7^K126R^ caused EGFR degradation inhibition (Figure 4A), which was further confirmed in patient fibroblasts (Figure 4C). In addition, the total EGFR amount was strongly increased in the RAB7^K126R^ patient compared to control fibroblasts (Figure 4B). These findings were confirmed by WB on the sural nerve (Figure 5A) and immunohistochemistry on skin biopsy which showed increased EGFR staining in the proband compared to the control although primarily in the surround of nerve fibers (Figure 5B). Three hours after internalization most of EGFR was still in early endosomes in patient cells, demonstrating that impaired EGFR trafficking to late endosomes and lysosomes causes inhibition of degradation and accumulation of EGFR (Figure 4D,E).

It is tempting to speculate that the prominent motor symptoms observed in our patients might be related to the differences in EGFR amount and degradation rate in contrast with previously studied CMT2B mutants associated with the typical sensory-predominant phenotype. EGF signaling mediated by EGFR is important for development and maintenance of various tissues including the nervous system, and EGFR is expressed in differentiated post-mitotic neurons suggesting pleiotropic functions, although the possibility for this receptor to exert distinct actions on different kind of neurons (e.g., motor versus sensory) is still unclear. Besides its key role in regulating neural stem cell proliferation, self-renewal, differentiation, and migration, EGF has neurotrophic and neuromodulatory functions on different kinds of neurons, increasing neuronal survival [31]. EGFR signaling is also required for proper cutaneous innervation during development and it seems to limit axonal outgrowth and branching [44]. Interestingly, after central nervous system (CNS) injuries and diseases, activation of the EGFR pathway triggers quiescent astrocytes to become reactive and destructive to neurons [45,46]. After spinal cord injury, EGFR activates astrocytes that represent a physical barrier to axon regeneration, expressing and secreting molecules that inhibit nerve growth [47]. Consistently, rats subjected to weight-drop spinal cord injury treated with a potent EGFR inhibitor to the injured area show motor and sensory function improvement [48]. Lack of EGFR expression is associated with progressive neurodegenerative disorders in mice [49,50]. Overexpression of EGFR is associated with cancer (high-grade glioma) [51]. Moreover, EGFR has a crucial role in the neurometabolic axis seen in the aging process [52]. Glia and endothelial cells demonstrate induced expression of EGFR after an acute injury or chronic neurodegeneration and there are multiple and mechanistically diverse routes by which the EGFR may be involved in the neuronal aspect of aging-related disorders and neurodegeneration. Consistently, it has been demonstrated that inhibition of EGFR enhances peripheral nerve regeneration after injury [30,53,54].

Thus, the inhibited degradation and the consequent accumulation of EGFR observed in RAB7^K126R^ cells could cause an increase in EGFR signaling leading to inhibition of axonal regeneration. The above-mentioned absence of nerve regeneration at nerve biopsy in our patient is of interest in this context.

EGFR has also been implicated in the expansion of the peripheral nervous system (PNS) progenitor cells, including Schwann cell precursors [55]. After peripheral nerve injury, EGFR expression is upregulated and promotes proliferation and migration of Schwann cells [56]. Importantly, EGFR hyperactivation induces a peripheral neurodegenerative disease as transgenic mice overexpressing the EGFR ligand epigen show a phenotype similar to CMT1A and CMT4F animal models [57].

Although there are at present no data regarding a specific role of EGFR in motoneurons, literature data on EGF signaling and EGFR in both CNS and PNS, suggest that the inhibition of EGFR degradation could be a possible explanation for the different clinical presentation in our family compared to the previously studied CMT2B families.

Recently, Wong et al. showed a role for RAB7 in driving the interactions between lysosomes and mitochondria, opening a new field of investigations for unraveling RAB7 functions [16]. We did not find any evidence of mitochondrial abnormalities in the proband’s sural nerve, but it will be interesting to study the role of mitochondria in CMT2B in the future and to verify whether these organelles play a key role in the phenotype and its variants.

## 5. Conclusions

We demonstrated that the behavior of the RAB7^K126R^ protein was similar to the previously studied CMT2B-causative RAB7 mutated proteins in all investigated functions except EGFR degradation, as RAB7^K126R^ showed inhibited degradation of the receptor. It is tempting to speculate that such difference might explain the distinctive clinical presentation in this family, but further studies are needed. This report expands the phenotypic spectrum of CMT2B: *RAB7* has to be added to the list of genes causing the classic CMT2 phenotype with motor predominance and must be considered in the genetic work-up of these patients.

## Figures and Tables

**Table 1:** *RAB7A* mutations associated with Charcot–Marie–Tooth type 2B (CMT2B).

Mutation	Clinical Phenotype	Families/Sporadic Cases	Age of Onset	Reference
L129F	CMT2B ulcero-mutilating features	Three related Austrian families	Adolescence—adulthood	[1,8]
K157N	CMT2B ulcerations, osteomyelitis, amputation	A patient (*de novo* mutation)	Adolescence	[3]
N161I	CMT2B pain, no muscle atrophy, and (except for the proband) ulcero-mutilating features	A Chinese family	Teens or later	[5]
N161T	CMT2B pain, ulcers, gangrene, and amputations	An English family	Adolescence	[2]
V162M	CMT2B ulcero-mutilating features (except for pt I-2 of the Scottish family who had drop feet but probably no ulcers)	Five unrelated families (from USA, Scotland, Austria, Belgium, Italy)	Adolescence—adulthood	[1,4,7,9,10]
K126R	CMT2 Predominantly motor phenotype with little sensory involvement	One Italian family	Adolescence	Present paper

**Table 2:** Clinical features of patients.

**Patients**	**Proband**	**Father**
Age at examination, yrs.	39	55
Onset age/symptoms	14/Cramps, gait difficulties	11/Gait difficulties
Motor symptoms legs	Complete footdrop, AFOs	Complete footdrop, AFOs
Sensory symptoms legs	Slight sensory loss feet	N
Proximal/Distal weakness UL	−/+- (4+; 4+)	−/+ (2;1)
Proximal/Distal weakness LL	−/+ (0;3)	−/+ (0;0)
Ankle deep tendon reflexes	Absent	Absent
Pinprick UL/LL	Normal/Ankles	Normal/Normal
Joint position sense UL/LL	Normal/Normal	Normal/Toes
Vibration sense UL/LL	Normal/Toes	Normal/Ankles
Pes cavus	N	Y
Additional features	Mild toenail dystrophy	Hand tremor
Ulnar nerve SAP amplitude	5.9 µV (ulnar nerve)	0.8 µV (median nerve)
CMTES	9/28	11/28

Motor weakness based on Medical Research Council Scale: upper limb distal weakness assessed by first dorsal interosseous and abductor pollicis brevis; upper limb proximal weakness assessed by deltoids, biceps brachii and triceps. Lower limb distal weakness assessed by anterior tibialis and gastrocnemius; lower limb proximal weakness assessed by iliopsoas and quadriceps muscles. Values are based on the side that gave the worst score. Abbreviations: AFO = ankle-foot orthosis; UL = upper limbs; LL = lower limbs; N = no; Y = yes; SAP = sensory action potential; CMTES = Charcot–Marie–Tooth examination score; + = weakness present; - = weakness absent; +- = mild weakness.

## Supplementary Materials

The following are available online at https://www.mdpi.com/2073-4409/9/4/1028/s1, Figure S1: Endocytosis pathway as shown by the KEGG database (modified) [58,59,60], Table S1: List of genes analyzed (Charcot–Marie–Tooth disease and related disorders), Table S2: Rare genetic variants (MAF < 1%) found in the analyzed genes of the proband, Table S3: Common genetic variants (MAF ≥ 1%) found in the analyzed genes of the proband.

## List of Abbreviations

AFO: ankle-foot orthosis; CMAP: compound muscle action potential; CMT2B: Charcot–Marie–Tooth type 2B; CMTES/CMTNSv2: CMT Examination/Neuropathy Score version 2; CNS: central nervous system; DTT: dithiothreitol; EEA1: early endosome antigen 1; EGFR: epidermal growth factor receptor; ESCRT-II: endosomal sorting complex required for transport; HA: hemagglutinin; HRP: horseradish peroxidase; HSANs: hereditary sensory and autonomic neuropathies; IENF: intraepidermal nerve fiber; MAF: minor allele frequency; MBP: myelin basic protein; MRC: Medical Research Council; NCS: nerve conduction studies; NF-H: high molecular weight neurofilament; MTOC: microtubule organizing center; NGS: Next Generation Sequencing; NCV: nerve conduction velocities; PGP9.5: protein gene product 9.5; PNS: peripheral nervous system; PTEN: phosphatase and tensin homolog; RAB7A: Ras-related in brain 7a; RILP: RAB-interacting lysosomal protein; SAP: sensory action potential; TBC1D15: Tre-2-Budding Uninhibited by Benzimidazole-Cell Division Cycle 16 Domain, Domain Family Member 15; TrkA: Tropomyosin receptor kinase A; VIP: vasoactive intestinal peptide; VPS22: vacuolar-sorting protein 22.
