# Supplementary material for: Charcot-Marie-Tooth Type 2B: A New Phenotype Associated with a Novel RAB7A Mutation and Inhibited EGFR Degradation
Source: Cells. 2020 Apr 21;9(4):1028. doi: 10.3390/cells9041028 (PMC7226405; doi:10.3390/cells9041028)
Supplement: Supplementary file 1 [file cells-09-01028-s001.zip › Table S1.pdf]

Table S1. List of genes analyzed (Charcot-Marie-Tooth disease and related disorders).

**Disease/Subpanel**	**Genes**
Charcot-Marie-Tooth type 1	EGR2,FGD4,FIG4,GDAP1,GJB1,GJB1_Promoter,LITAF,MPZ,MTMR2, NDRG1,NEFL,PMP22,PRX,SBF2, SH3TC2
Charcot-Marie-Tooth type 2	AARS,BSCL2,DNM2,DYNC1H1,GARS,GDAP1,GJB1,GJB1_Promoter,HINT1, HSPB1,HSPB8,IGHMBP2,LMNA,LRSAM1,MARS,MFN2,MPZ,NEFL,PMP22, PRPS1,RAB7A,TRPV4,YARS
Distal hereditary motor neuropathies	ATP7A,BICD2,BSCL2,DCTN1,DYNC1H1,GARS,HSPB1,HSPB3,HSPB8,IGHMBP2, SETX,SLC52A1,SLC52A2,SLC52A3,TRPV4
Hereditary sensory neuropathies	ATL1,CCT5,FAM134B,NGF,NTRK1,RAB7A,SCN9A,SPTLC1,SPTLC2,WNK1
Hereditary spastic paraplegias	CYP7B1,FA2H,GJC2,HSPD1,KIAA0196,KIF5A,NIPA1,PLP1,PNPLA6,REEP1,RTN2, SPAST,SPG7
Mitochondrial DNA depletion syndromes/ Progressive external ophthalmoplegia	C10orf2,DGUOK,MFN2,MPV17,OPA1,POLG,POLG2,RRM2B,SLC25A4,SUCLA2, SUCLG1,TK2,TYMP
