# Supplementary material for: Charcot-Marie-Tooth Type 2B: A New Phenotype Associated with a Novel RAB7A Mutation and Inhibited EGFR Degradation
Source: Cells. 2020 Apr 21;9(4):1028. doi: 10.3390/cells9041028 (PMC7226405; doi:10.3390/cells9041028)
Supplement: Supplementary file 1 [file Figure S1.pdf › Figure S1.pdf]

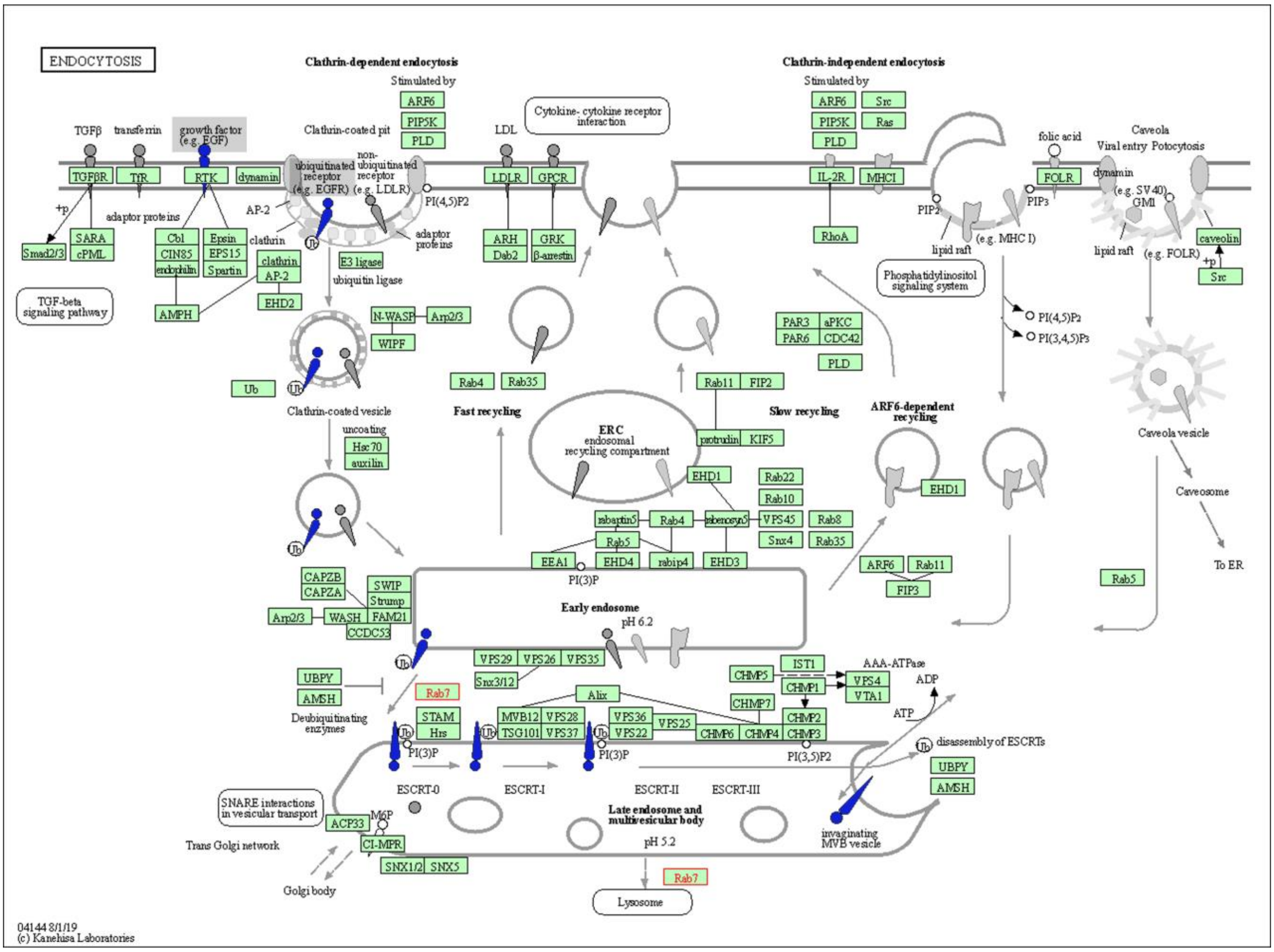

Figure S1. Endocytosis pathway as shown by the KEGG database (modified) [58-60]. EGF and EGFR in the pathway are depicted by grey boxes and colored in blue. The RAB7 protein is highlighted in red. Arrows depict signaling routes.
